# A Weekly Session of Jumping Interval Training Effectively Enhances Aerobic, Anaerobic, and Jumping Performance in Aerobic Gymnastics

**DOI:** 10.5114/jhk/193536

**Published:** 2024-12-19

**Authors:** Dong Ma, Ke Wang, Rui Miguel Silva, Qi Xu, Zijian Zhao

**Affiliations:** 1 Department of Biomecahnics, Gdansk University of Physical Education and Sport, Gdańsk, Poland.; 2 School of Sport Education, Tianjin University of Sport, Tianjian, China.; 3 School of Sport and Leisure, Polytechnic Institute of Viana do Castelo, Viana do Castelo, Portugal.; 4 Sport Physical Activity and Health Research & Innovation Center, Viana do Castelo, Portugal.; 5 School of Physical Education, Zhengzhou University Headquarters, Henan, China.

**Keywords:** gymnastics, athletic performance, strength and conditioning, physical exercise, sports training

## Abstract

This study aimed to compare the impact of varying weekly frequencies of jumping interval training (JIT) on aerobic and anaerobic fitness, as well as jumping abilities of youth female athletes specialized in aerobic gymnastics. A randomized controlled study design was conducted spanning 8-week duration, involving 69 youth female athletes (16.3 ± 1.2 years) specialized in aerobic gymnastics. Participants were allocated into two experimental groups: JITw1 (comprising individuals subjected to JIT once a week), and JITw2 (encompassing individuals undergoing JIT twice a week), alongside a control group. Prior to and post the intervention period, athletes underwent evaluations of their performance through the countermovement jump test (CMJ), a specialized anaerobic assessment personalized for aerobic gymnasts (SAGAT), and a 20-m multistage fitness test. A mixed ANOVA was conducted for statistical analysis. Significant time (baseline and post-intervention) x group (JITw1, JITw2 and control) interactions were found in the SAGAT (p < 0.001), the CMJ (p < 0.001) and the 20-m multistage fitness test (p < 0.001). Post-intervention analysis revealed significantly lower scores in the SAGAT for the control group compared to the JITw2 group (p = 0.003). Significantly higher scores were observed for the JITw2 group in the CMJ test compared to the control group (p = 0.001). Significantly lower scores in the 20-m multistage fitness test were found in the control group compared to the JITw2 and JITw1 groups (both p < 0.001). As conclusion, while additional JIT training once a week may suit for minimal effective training and positive adaptations, training twice a week is advisable when significant improvements are desired.

## Introduction

Aerobic gymnastics imposes significant demands on the body's cardiorespiratory and metabolic systems (Aleksandraviciene et al., 2015). These demands arise from the need to sustain intermittent, high-intensity routines, which heavily engage both aerobic and anaerobic pathways (Kyselovičová and Danielová, 2012; Mikołajec et al., 2023). Athletes are required to possess well-developed cardiovascular fitness to meet these physiological demands (Cuce et al., 2021). Studies indicate that athletes competing at higher levels demonstrate greater reliance on anaerobic energy sources (Aleksandraviciene et al., 2015). This underscores the importance of not just mastering movement skills, but also executing routines with high intensity and complexity (Aleksandraviciene et al., 2015). In addition to cardiovascular fitness, success in aerobic gymnastics is dependent on muscular strength and strength endurance (Budiarti et al., 2022). Athletes must be capable of effectively performing dynamic and ballistic movements across various muscle groups (Puiu and Dragomir, 2020). Thus, achieving peak performance in this sport requires exceptional balance between energy systems, physiological adaptations, and muscular capabilities (Fischerova et al., 2012; Lamošová et al., 2021; Zhao et al., 2023).

By considering the physical demands of the sport, high-intensity interval training (HIIT) can be a highly effective method for enhancing the aerobic and anaerobic capacities of aerobic gymnastics athletes (Buchheit and Laursen, 2013a, 2013b). Aerobic improvements are facilitated by HIIT ability to elevate maximal oxygen uptake (Gibala and McGee, 2008), a key determinant of aerobic capacity. Moreover, HIIT enhances anaerobic capacity (Abe et al., 2015) through improvements in the lactate threshold and the buffering capacity, crucial for sustaining high-intensity efforts characteristic of aerobic gymnastics performances. Mechanistically, HIIT induces mitochondrial biogenesis and improves muscle oxidative capacity, enabling more efficient energy production and utilization (Hoshino et al., 2016).

HIIT is commonly structured into four primary regimens (Buchheit and Laursen, 2013b): short intervals, involving sub-maximal running efforts performed at >100% of maximal aerobic speed, with intervals lasting less than 60 s; long intervals, characterized by sub-maximal efforts performed between 85 and 100% of maximal aerobic speed, with intervals lasting between 120 and 240 s; repeated sprint training, involving maximal efforts lasting less than 10 s, with short recovery intervals in between; and sprint interval training, comprising maximal efforts with longer rest intervals between repetitions. While HIIT is a widely-used method, its most popular modalities are typically associated with running or cycling. However, the unique demands of aerobic gymnastics necessitate alternative modalities that closely mimic the comprehensive impact of multiple ballistic movements, frequently involving repeated jumps. An effective strategy worth considering involves the integration of jumping interval training (JIT) protocols (Kramer et al., 2019). These protocols are specifically designed to improve muscular endurance and power while also targeting enhancements in metabolism and the underlying bioenergetic systems crucial for the sport. JIT entails alternating between bursts of high-intensity effort and periods of recovery (Ache-Dias et al., 2016). This approach not only supports both aerobic and anaerobic capacities, but also can positively impact muscle endurance and power, essential for coping with the demands in aerobic gymnastics (Ache-Dias et al., 2016).

While the potential benefits of JIT to aerobic and anaerobic performance, as well as jumping ability among gymnastic athletes, are promising, empirical evidence in this realm remains limited (Abuwarda et al., 2024; Afroundeh et al., 2020). For instance, a study involving thirty novice children found that a regimen combining continuous jumping with anaerobic training led to evident improvements in maximal oxygen uptake (Afroundeh et al., 2020). In another investigation, HIIT utilizing muscular endurance exercises demonstrated significant efficacy in enhancing specific shuttle run and vertical jump performances among youth gymnasts (Abuwarda et al., 2024).

Despite the limited evidence regarding the application of JIT methodology in sports training, particularly in aerobic gymnastics, the effects of training volume, including frequency and the total number of repetitions, on the adaptations induced by this training method remain unknown. Implementing these methods may pose challenges in terms of space allocation within weekly training schedules. Therefore, identifying the most effective dosage within a minimal training time could be particularly beneficial for enhancing coach availability to implement these strategies. Although studies focusing on gymnastic athletes are lacking, it has been observed that a minimum of 13 HIIT sessions seems sufficient to ensure significant improvements in aerobic capacity in sedentary populations (Weston et al., 2014). However, information specific to athletes is scarce, highlighting the need for studies comparing lower versus higher training frequency and volume of JIT. Such studies may help identify the minimal dosage required to observe significant adaptive changes in athletes. This not only benefits the current state of the art, but also enables to optimize the implementation of effective protocols within the busy schedules of gymnastics training. Considering the aforementioned reasons, this study aimed to compare the effects of one weekly session versus two weekly sessions of JIT on aerobic and anaerobic fitness, as well as jumping abilities of young female athletes specialized in aerobic gymnastics.

## Methods

### Participants

The sample size was determined a priori with G*Power software (version 3.1.9, Universität Düsseldorf, Germany), targeting an effect size of 0.2 across three groups and two measurement points. This approach aimed to secure a statistical power of 0.85, maintaining a significance level of 0.05 for F-tests, specifically tailored for ANOVA analyses involving repeated measures and interactions between and within groups. Based on this calculation, software recommended enrolling 72 participants.

The following inclusion criteria were applied: (i) attendance at both assessment points, (ii) a minimum of two years' experience in aerobic gymnastics, (iii) participation in at least 85% of standard training sessions, (iv) absence of injury or illness during the experiment and the preceding month, (v) exclusive enrollment in aerobic gymnastics training without additional programs, and (vi) being female.

**Figure 1:**
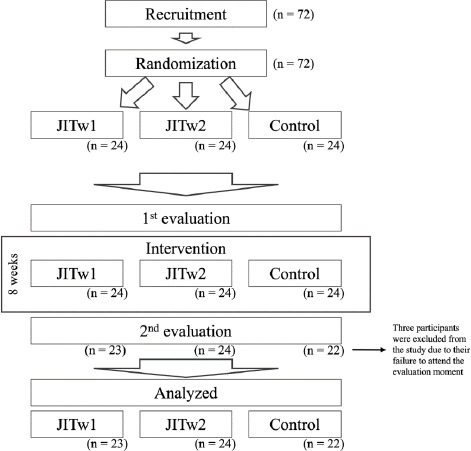
Participants flow chart.

Seventy-two participants were initially recruited and incorporated into the study. However, due to non-adherence to the requirement of being evaluated at both time points (stemming from their unavailability during the assessment week), only 69 participants were ultimately analyzed (Figure1).

In total, the study involved 69 female participants, with an average age of 16.3 years (standard deviation ± 1.2), body height of 1.61 m (± 0.03), and body mass of 52.7 kg (± 2.3). The breakdown of anthropometric and demographic data by groups is detailed in Table 1. Participants engaged in 4 to 5 weekly training sessions and competed at the regional level. Participants hailed from three distinct clubs, all operating under the same training approach and competitive level. To ensure equitable distribution, participants from each club were allocated to all three groups. Consequently, Club A contributed 18 athletes (JITw1 = 6; JITw2 = 6; Control = 6), Club B contributed 24 athletes (JITw1 = 8; JITw2 = 8; Control = 8), and Club C contributed 27 participants (JITw1= 9; JITw2 = 9; Control = 9).

**Table 1:** Mean ± standard-deviation of the demographic and anthropometric information for groups.

	JITw1 (n=23)	JITw2 (n=24)	Control (n=22)
Age (y)	16.3 ± 1.2	16.1 ± 1.1	16.4 ± 1.2
Body height (m)	1.61 ± 0.03	1.60 ± 0.04	1.61 ± 0.03
Body mass (kg)	52.7 ± 2.4	52.8 ± 2.4	52.5 ± 2.2
Body mass index (kg/m^2^)	20.3 ± 0.7	20.6 ± 0.7	20.3 ± 0.5

JITw1: jumping interval training (one weekly training session); JITw2: jumping interval training (two weekly training sessions)

Gymnasts and their parents or legal guardians received detailed information about the study's protocol and background. Upon voluntarily agreeing to participate, the legal guardians provided their signature on an informed consent form. This study obtained approval from the Ethics Committee of the Tianjin Institute of Physical Education (protocol code: TJUS2024/016; approval date: 02 January 2024) and adhered to the ethical standards outlined in the Declaration of Helsinki for research involving human subjects.

### Measures

#### Anthropometrics

Standard anthropometric assessments were applied to determine participants' body height and mass. Body height measurements were taken utilizing a stadiometer (Seca 217, Seca, Hamburg), while body mass was recorded using an electronic scale (SECA 813; Seca GmbH & Co., Hamburg, Germany) with precision of 0.1 kg. Participants wore leotards during measurements to maintain consistency.

#### Countermovement Jump

Participants were directed to execute three maximal countermovement jumps (CMJs), with a designated 1-min rest interval between each jump. They were instructed to maintain extended knees and hands on hips during the jump, followed by controlled landing on the floor. CMJ performance was assessed using the MyJump 2 mobile application (version 1.0.8), employing video analysis to determine flight time and estimate jump height. Previous research (Haynes et al., 2019) had validated the accuracy and precision of this application. The smartphone used was an iPhone 13 (Apple Inc.), positioned securely in a mount at a height of 50 cm, with its orientation fixed against a white wall. Athletes consistently jumped from the same spot, marking a square on the floor, displaced 2 m from the smartphone. The average jump height derived from the three attempts was registered for subsequent data analysis.

#### Specific Aerobic Gymnastics Anaerobic Test

The Specific Aerobic Gymnastics Anaerobic Test (SAGAT) entails executing gymnastics-specific maneuvers in a maximal repeated sprint format within a prescribed timeframe of 80–90 s (Alves et al., 2015). It consists of two sets, each comprising six consecutive bouts. Each bout involves a “tuck jump”, succeeded by a drop to perform two “push-ups” and one “L-support”. Validity against the Wingate test was established, and reliability was confirmed, affirming it as a precise and dependable measure of anaerobic performance in female aerobic gymnasts (Alves et al., 2015). Researchers monitored participants' execution of test elements and controlled the execution time using a digital stopwatch integrated into the iPhone camera software. SAGAT performances were recorded using an iPhone, and the starting and ending points were selected to determine the duration of actions. Test completion time (measured in seconds) served as the basis for subsequent data analysis.

#### 20-m Multistage Fitness Test

Participants engaged in the 20-m multistage fitness test (Léger et al., 1988), a commonly employed method for evaluating the aerobic capacity of gymnasts (Salse-Batán et al., 2022). The test commenced at an initial speed of 8.5 km/h, with the frequency of audio signals increasing the speed by 0.5 km/h every minute until participants failed to reach the designated markers upon hearing the signal. Termination of the test occurred when participants were unable to meet the marker for the second consecutive time due to fatigue. Aerobic performance was quantified by measuring the total distance covered in meters.

### Design and Procedures

The research adopted a single-blind randomized controlled design, introducing two experimental groups (JITw1 and JITw2) alongside the usual training routines. There was also a control group that continued with their standard aerobic gymnastics regimen. Gymnasts were selected for participation from aerobic gymnastics teams through a convenience sampling method. To ensure the randomness of group assignments, a pre-assessment randomization procedure was employed, utilizing opaque envelopes that were distributed to the gymnasts prior to their initial evaluation. Assessments were conducted one week prior to the start of the interventions and immediately following the conclusion of the 8-week period.

The assessment procedures were structured and executed to ensure consistency throughout the study. The evaluations conducted before and after the intervention period occurred on identical weekdays. Indoor settings were chosen to maintain controlled conditions. To minimize variability, evaluations were scheduled after a 48-h rest period post-training. The sequence of evaluations followed a systematic approach: (i) demographic data collection, (ii) anthropometric measurements, (iii) a structured warm-up routine comprising 5 min of running, 15 min of dynamic stretching, and 5 min of jumping drills, (iv) execution of three repetitions of countermovement jumps, (v) completion of the specific aerobic gymnastics anaerobic test, and (vi) participation in the 20-m multistage fitness test. A standardized 5-min rest interval separated each assessment to mitigate fatigue effects. All participants underwent evaluations in the same sequential order during both pre- and post-intervention periods. It is also important to note that athletes were familiarized with the tests earlier in the season, during the first week of their training sessions. These tests were the same ones used for their club assessments. This early introduction allowed athletes to learn the proper techniques, understand the movement patterns, and meet the requirements of the tests.

### Jumping Interval Training

While the control group just engaged in standard training sessions, participants assigned to either JITw1 or JITw2 underwent one or two additional sessions per week applying JIT. The training process took place early in the season, three weeks after the start of training sessions. Each regular session generally ranged from 80 to 120 min and encompassed a variety of warm-up exercises, both general and specific. Warm-up activities comprised static and dynamic stretching, alongside multi-jump exercises, leading into a focused strength and conditioning component primarily aimed at enhancing muscular endurance. The primary focus of training circled around refining technical skills and gymnastics routines. Finally, each session concluded with a cooldown period centered on stretching exercises.

These supplementary sessions preceded regular training. Before each JIT session, participants followed a standardized warm-up protocol, including 5 min of running, 15 min of dynamic stretching, and 5 min of jumping drills. With regard to JITw1, this session occurred before the first training session of the week. For JITw2, sessions were conducted before both the first and fourth training sessions of the week, with a 48-h interval between sessions.

Both JIT groups underwent identical training sessions, with participants in JITw2 completing double the session frequency, thereby increasing the weekly training volume. The JIT protocol consisted of five sets of maximal continuous bilateral countermovement jumps lasting 30 to 40 s each, as detailed in Table 2. Participants were guided by an audio beep to maintain a pace of 1.1 to 1.0 jumps per second, following recommendations from previous studies (Ache-Dias et al., 2016; Kramer et al., 2019). A 30-s rest period separated each bout. This methodology was based on prior research suggesting optimal work duration of 30 to 40 s for maximal continuous jumping, as well as specific rest intervals to effectively target both the aerobic and the anaerobic metabolism (Ache-Dias et al., 2016; Kramer et al., 2019).

**Table 2:** Description of the training interventions using jumping interval training.

Week	Session	JITw1	JITw2
1	1	4×30’’:30’’ at 1.1 jump/s	4×30’’:30’’ at 1.1 jump/s
1	2	NA	4×30’’:30’’ at 1.1 jump/s
2	3	4×30’’:30’’ at 1.1 jump/s	4×30’’:30’’ at 1.1 jump/s
2	4	NA	4×30’’:30’’ at 1.1 jump/s
3	5	4×40’’:30’’ at 1 jump/s	4×40’’:30’’ at 1 jump/s
3	6	NA	4×40’’:30’’ at 1 jump/s
4	7	4×40’’:30’’ at 1 jump/s	4×40’’:30’’ at 1 jump/s
4	8	NA	4×40’’:30’’ at 1 jump/s
5	9	5×30’’:30’’ at 1.1 jump/s	5×30’’:30’’ at 1.1 jump/s
5	10	NA	5×30’’:30’’ at 1.1 jump/s
6	11	5×30’’:30’’ at 1.1 jump/s	5×30’’:30’’ at 1.1 jump/s
6	12	NA	5×30’’:30’’ at 1.1 jump/s
7	13	5×40’’:30’’ at 1 jump/s	5×40’’:30’’ at 1 jump/s
7	14	NA	5×40’’:30’’ at 1 jump/s
8	15	5×40’’:30’’ at 1 jump/s	5×40’’:30’’ at 1 jump/s
8	16	NA	5×40’’:30’’ at 1 jump/s

JITw1: jumping interval training (one weekly training session); JITw2: jumping interval training (two weekly training sessions); NA: not applicable

### Statistical Analysis

Before proceeding with inferential statistics, the normality of the sample distribution was evaluated and confirmed through the Kolmogorov-Smirnov test (*p* > 0.05), while homogeneity assumption was confirmed using the Levene’s test (*p* > 0.05). Considering the study's design involving two assessments across three groups, a mixed ANOVA was utilized to examine interactions between time and groups. This analysis also involved the computation of partial eta squared (${\text{η}}_{p}^{2}$). The effect size magnitude was categorized as follows (Richardson, 2011): trivial (< 0.01), small (0.01–0.06), moderate (> 0.06–0.14), and large (> 0.14). Furthermore, post-hoc comparisons were carried out employing the Bonferroni test. To compare the improvements represented by the delta post-pre (calculated using the formula post-pre/pre*100) for each outcome, a one-way ANOVA was conducted, followed by a Bonferroni post hoc test. Statistical analyses were executed utilizing JASP software (version 0.18.3, University of Amsterdam, The Netherlands), with a predefined significance level of *p* < 0.05.

## Results

Comparisons between groups revealed no significant differences in baseline results of the SAGAT (*F*_2,66_ = 0.405; *p* = 0.669; ${\text{η}}_{p}^{2}$ = 0.012), the CMJ (*F*_2,66_ = 0.775; *p* = 0.465; ${\text{η}}_{p}^{2}$ = 0.023) and the 20-m multistage fitness test (*F*_2,66_ = 2.313; *p* = 0.107; ${\text{η}}_{p}^{2}$ = 0.066).

Significant time (baseline and post-intervention) × group (JITw1, JITw2 and control) interactions were found in the SAGAT (*F*_2,66_ = 33.635; *p* < 0.001; ${\text{η}}_{p}^{2}$ = 0.505), the CMJ (*F*_2,66_ = 10.216; *p* < 0.001; ${\text{η}}_{p}^{2}$ = 0.236) and the 20-m multistage fitness test (*F*_2,66_ = 11.475; *p* < 0.001; ${\text{η}}_{p}^{2}$ = 0.258).

**Figure 2:**
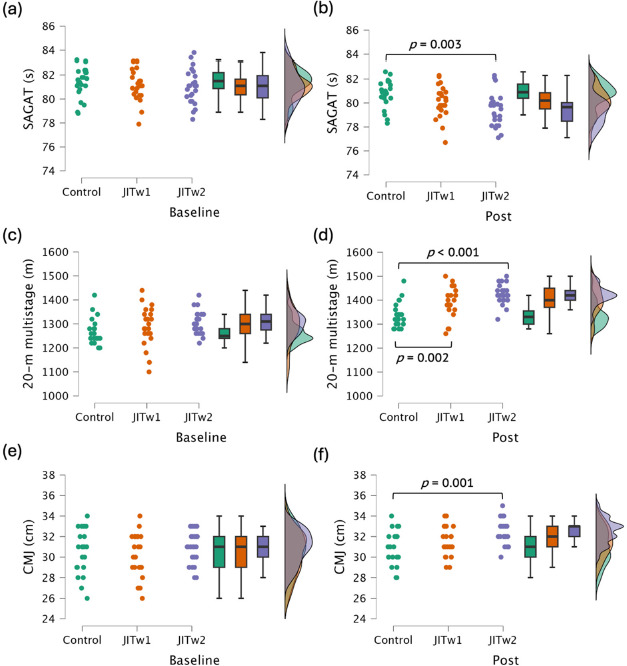
Between-group comparisons in the specialized anaerobic assessment test (SAGAT) (a: baseline, b: post-intervention), the 20-meter multistage fitness test (c: baseline, d: post-intervention), and the countermovement jump (CMJ) test (e: baseline, f: post-intervention). JITw1: jumping interval training (one weekly training session); JITw2: jumping interval training (two weekly training sessions); boxplots: the line dividing the box into two halves represents the median value. This indicates that 50% of the data points are below this median value, and the other 50% are above it. The left edge of the box represents the lower quartile, indicating the point where the first 25% of the data fall. Similarly, the right edge of the box represents the upper quartile, indicating where the top 25% of the data lie. The ends of the horizontal lines signify the maximum and minimum values of the dataset.

The time × group analysis post-intervention revealed significantly lower scores in the SAGAT for the control group compared to the JITw2 group (−1.322 s; *p* = 0.003). Additionally, significantly higher scores were observed for the JITw2 group in the CMJ test compared to the control group (+1.587 cm; *p* = 0.001). Post-intervention revealed significantly lower scores in the 20-m multistage fitness test for the control group compared to the JITw2 (−85.985 m; *p* < 0.001) and the JITw1 group (−57.470 m; *p* < 0.001). Figure 2 displays the graphical comparisons between groups.

**Figure 3:**
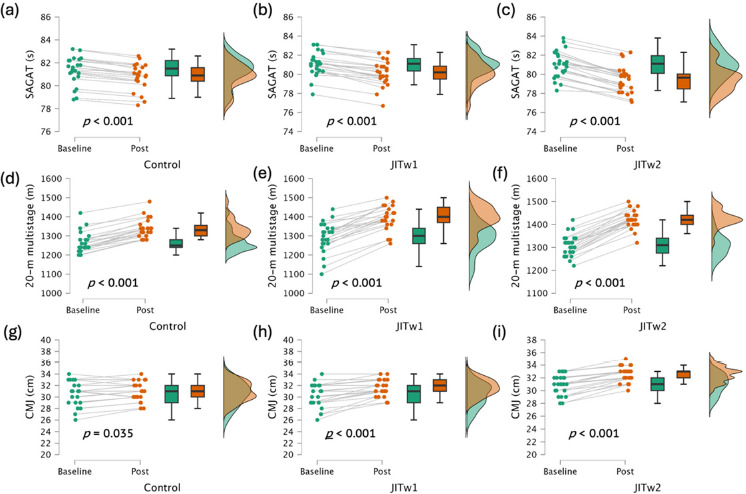
Within-group comparisons in the specialized anaerobic assessment test (SAGAT) (a: the control group, b: the JITw1 group, and c: the JITw2 group), the 20-meter multistage fitness test (d: the control group, e: the JITw1 group, and f: the JITw2 group) and the countermovement jump (CMJ) test (g: the control group, h: the JITw1 group, and i: the JITw2group). JITw1: jumping interval training (one weekly training session); JITw2: jumping interval training (two weekly training sessions). Boxplots: the line dividing the box into two halves represents the median value. This indicates that 50% of the data points are below this median value, and the other 50% are above it. The left edge of the box represents the lower quartile, indicating the point where the first 25% of the data fall. Similarly, the right edge of the box represents the upper quartile, indicating where the top 25% of the data lie. The ends of the horizontal lines signify the maximum and minimum values of the dataset.

Figure 3 displays the graphical comparisons within groups. The JITw2 group significantly improved scores in the SAGAT (–1.554 s; *p* < 0.001), the CMJ (+1.542 cm; *p* < 0.001), and the 20-m multistage fitness test (+116.7 m; *p* < 0.001). Additionally, the JITw1 group significantly improved scores in the SAGAT (–0.952 s; *p* < 0.001), the CMJ (+1.40 cm; *p* < 0.001) and the 20-m multistage fitness test (+104.3 m; *p* < 0.001). Finally, the control group significantly improved performance in the SAGAT (–0.559 s; *p* < 0.001), the CMJ (+0.409cm; *p* = 0.035) and the 20-m multistage fitness test (+70.0 m; *p* < 0.001).

**Figure 4:**
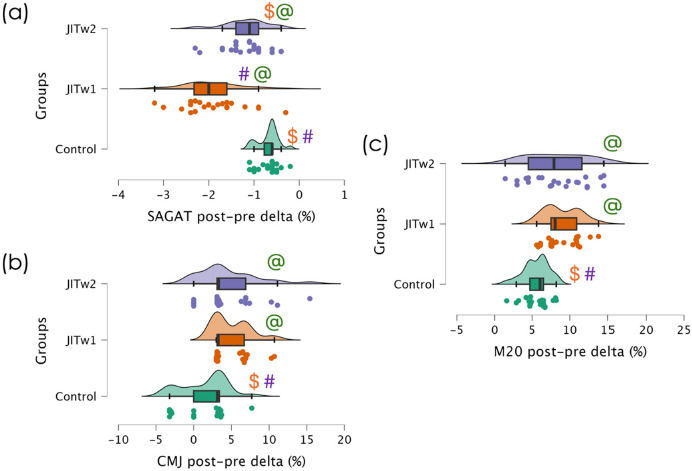
Comparison of the delta (percentage change post-pre) for each outcome. (a) SAGAT: specialized anaerobic assessment test; (b) CMJ: Countermovement jump test; (c) M20: the 20-m multistage fitness test. *JITw1: jumping interval training (one weekly training session); JITw2: jumping interval training (two weekly training sessions); @: significantly different from the control group (p < 0.05); $: significantly different from the JITw1 group (p < 0.05); #: significantly different from the JITw2 group (p < 0.05)*

Figure 4 illustrates comparisons among groups regarding the within-group percentage differences, thereby assessing the improvements within each group. Significant differences were identified between groups for the SAGAT delta (*F* = 35.810; *p* < 0.001; ${\text{η}}_{p}^{2}$ = 0.520), the CMJ (*F* = 8.277; *p* < 0.001; ${\text{η}}_{p}^{2}$ = 0.201), and the multistage fitness test (*F* = 9.073; *p* < 0.001; ${\text{η}}_{p}^{2}$ = 0.216). Regarding the SAGAT, significantly greater improvements were observed in JITw1 (*p* < 0.001) and JITw2 (*p* = 0.003) groups compared to the control group, with differences also noted between the JITw1 and the JITw2 group (*p* < 0.001). For the CMJ, significantly greater improvements occurred in the JITw1 (*p* < 0.001) and the JITw2 group (*p* = 0.006) compared to the control group. Lastly, in the multistage fitness test, significantly greater improvements were observed in the JITw1 (*p* < 0.001) and JITw2 groups (*p* = 0.006) compared to the control group.

## Discussion

The current study aimed to compare the effects of one weekly session versus two weekly sessions of JIT on aerobic and anaerobic fitness, as well as jumping abilities of young female athletes specialized in aerobic gymnastics. Our findings suggest that implementing JIT training once a week can have substantial and similarly beneficial impact compared to implementing it twice a week on enhancing aerobic and anaerobic fitness, as well as jumping performance of aerobic gymnastics athletes. However, it is crucial to note that while implementing JITw2 showed significant improvement over the control group across all outcomes, the same level of significance was not observed with JITw1. Nevertheless, there was no significant difference between JIT once and twice a week. These findings suggest that JIT once a week might provide a minimal effective dose, whereas for more pronounced adaptations, implementing JIT twice a week could be more advisable.

JIT, known as a form of HIIT, emerges as a noteworthy modality for improving anaerobic performance due to its acute effects. These effects can lead to pronounced reductions in lactate accumulation and an increased proportion of time spent above 90% of maximal oxygen uptake (Kramer et al., 2019). Previous research conducted on recreational runners has shown a significant improvement in anaerobic power and capacity through JIT (Ache-Dias et al., 2016), corroborating our own findings. Additionally, our study is consistent with prior investigations that have highlighted the beneficial impact of HIIT on anaerobic performance (Stöggl and Björklund, 2017). Several reasons can elucidate the beneficial impact of JIT. For instance our utilization of short-interval HIIT has been influential in elevating the body's lactate threshold. This enables athletes to tolerate higher levels of this metabolic byproduct, thereby delaying the onset of fatigue during anaerobic activities induced by the demanding high-intensity levels during exercise (Edge et al., 2006). Moreover, JIT may stimulate the production of enzymes involved in the anaerobic metabolism, such as lactate dehydrogenase, facilitating faster clearance of lactate and promoting efficient energy utilization (Little et al., 2010). Lastly, the neuro-muscular coordination required for jumping activities improves, leading to more efficient muscle recruitment patterns, further enhancing anaerobic performance (Vera-Ibañez et al., 2017).

Additionally, improvements in aerobic conditioning can enhance cardiovascular function, potentially increasing oxygen delivery to muscles (Rosenblat et al., 2022). This facilitates faster removal of metabolic by-products such as lactate during anaerobic efforts, thereby delaying the onset of fatigue and improving recovery between bouts of high-intensity exercise (Fiorenza et al., 2019). These enhancements in cardiovascular performance may also promote mitochondrial biogenesis and oxidative enzyme activity in muscle fibers (Hoshino et al., 2016). These adaptations improve the muscles' ability to utilize oxygen more efficiently and generate ATP through aerobic pathways (Torma et al., 2019). This indirect support can benefit anaerobic energy systems during prolonged or repeated bouts of anaerobic exercise.

Interestingly, one session per week showed to be similarly effective in enhancing anaerobic performance as two sessions, although the latter demonstrated significantly superior results compared to the control group. The fact that just one session suffices to yield improvements in anaerobic performance can be attributed to the intensity of the JIT regimen, which allows for intensities exceeding 90% of the maximal heart rate (Kramer et al., 2019). This intensity has been identified as the primary training stimulus of improvement as reported by previous research (Castagna et al., 2011; Runacres et al., 2019). However, it is noteworthy that the minimal effective dose within the JITw1 group was not significantly different from the control group, while JITw2 showcased significantly greater improvements compared to the control group. Therefore, while there was no significant difference between JITw1 and JITw2, it is prudent to suggest that engaging in twice-weekly sessions may exert a more pronounced influence on the magnitude of improvements.

Considering the impact of both JIT formats, significant improvements in the athletes' aerobic capacity were observed. Furthermore, both frequencies of training sessions (once or twice weekly) showed a significant advantage over the control group, with no significant difference between them. These findings corroborate previous research, indicating the effectiveness of jumping training in enhancing maximal oxygen uptake (Afroundeh et al., 2020), and showcasing improvements in specific shuttle run performance among youth gymnasts (Abuwarda et al., 2024).

By emphasizing heart rate responses and engaging in repeated jumps at maximum intensity, JIT demonstrates significant effectiveness in enhancing aerobic performance. This effectiveness stems from its potential positive influence on cardiac function (Astorino et al., 2012), such as the augmentation of stroke volume and cardiac output, resulting in improved oxygen delivery to active muscles (MacInnis and Gibala, 2017). Furthermore, JIT may have beneficial impact on mitochondrial biogenesis and oxidative enzyme activity within muscle fibers (Jacobs et al., 2013), consequently boosting the muscles' ability to utilize oxygen for energy generation (Little et al., 2011).

JITw1 can be similarly effective in improving aerobic capacity to JITw2 due to its ability to elicit substantial physiological adaptations in a shorter timeframe. JIT is expected to engage large muscle groups, demanding significant oxygen uptake and cardiovascular effort, thus stimulating cardiovascular adaptation (Arazi et al., 2012). This high-intensity activity may trigger improvements in cardiac output, stroke volume, and oxygen utilization efficiency (Astorino et al., 2012). Despite the reduced frequency, the intensity and specificity of JIT induce comparable improvements in aerobic capacity, making it a time-efficient alternative for coaches seeking performance enhancement (Yin et al., 2024).

Considering the influence of JIT on jumping performance as measured by the CMJ, it was noted that while the JITw2 group showed a significant improvement compared to the control group, the JITw1 group did not exhibit a significant difference from the JITw2 group, nor from the control group. It can be anticipated that JIT, involving maximal repeated countermovement jumps, may contribute to improved performance in the CMJ test due to the similarity of movement patterns and the heightened recruitment of muscles, accentuating the stretch-shortening cycle during continuous exercise (McCaulley et al., 2007). This mechanism amplifies the utilization of elastic energy stored in tendons, thereby facilitating increased force generation during jumps (Wade et al., 2018). Furthermore, the activation of fast-twitch fibers through maximal repeated jumps contributes significantly to increased strength gains (Potteiger et al., 1999), which are essential for enhancing jump height. However, although JITw1 effectively enhanced CMJ performance, it did not show a significant difference compared to the control group, whereas JITw2 did. This suggests that for situations demanding substantial improvements, incorporating two weekly JIT sessions may be necessary, while utilizing JIT once a week could suffice to ensure a minimal effective dose without significantly disrupting regular training schedules. However, future analysis could explore the impact of recovery between sets, as well as the pace and cadence of jumps within each set. Optimizing muscular recruitment during jumping might foster various adaptations (Venegas-Carro et al., 2023) that were not thoroughly explored in our study.

The present study offers valuable insights into the efficacy of implementing JIT once or twice a week on aerobic and anaerobic fitness, as well as jumping performance of aerobic gymnastics athletes. While our findings reveal significant improvements in anaerobic and aerobic capacities regardless of training frequency compared to the control group, some limitations and areas for future research emerge. Although JITw2 demonstrated superior results over the control group across all outcomes, the same level of significance was not observed with JITw1. This suggests that while JIT once a week may provide a minimal effective dose for some athletes, twice-weekly sessions could yield more pronounced adaptations. However, it is crucial to acknowledge that the lack of significant difference between JIT once and twice a week warrants further investigation into individual variability and potential confounding factors such as the accumulated external and internal training load. Furthermore, future research could investigate the enduring impacts of JIT on performance sustainability, examining factors such as trainability and plateau effects. Additionally, there is a need to explore the most effective timing and duration of JIT sessions within the training regimen of aerobic gymnastics athletes. Future studies should delve deeper into investigating the duration of the program and its correlation with the extension of training volume, as these factors warrant further research.

As study limitations, it is important to acknowledge that no external load measures were obtained during the intervention, which could have offered greater insight into the mechanisms associated with the observed improvements. Furthermore, incorporating more laboratory-based instruments to measure physiological mechanisms for explaining adaptations would be beneficial in enhancing the understanding of the impact of this training. Therefore, future research should incorporate external load monitoring during training sessions and include physiological tests focused on maximal oxygen uptake, the lactate threshold or the enzymatic profile to better comprehend the causes behind the observed adaptations.

Practical implications of our research findings suggest that implementing JIT once a week can yield substantial and comparable improvements in aerobic and anaerobic fitness, as well as jumping performance among aerobic gymnastics athletes, akin to implementing it twice a week. However, for scenarios requiring significant improvements, engaging in two JIT sessions per week may be preferable, while one session can serve as a minimal effective dose without significantly disrupting regular training schedules. These findings provide practical insights for optimizing training protocols and improving athletic performance in aerobic gymnastics.

## Conclusions

In conclusion, this study sheds light on the efficacy of JIT in enhancing aerobic, and anaerobic fitness, as well as jumping abilities of youth female athletes specialized in aerobic gymnastics. The findings reveal that both JITw1 and JITw2 resulted in significant improvements within groups across all measured variables. Particularly, the JITw2 group demonstrated superior outcomes compared to the control group in terms of anaerobic capacity, as evidenced by higher scores in the countermovement jump test and the 20-meter multistage fitness test. These results suggest that incorporating JIT into training regimens can yield substantial benefits for aerobic gymnasts, regardless of the frequency, with twice-weekly sessions offering a slight advantage in certain aspects. However, it should be noted that JITw1 still shows effectiveness, providing a minimal yet significant training dose that leads to positive improvements comparable to those seen with JITw2. Therefore, coaches and athletes alike may consider integrating JIT into their training routines to enhance overall aerobic and anaerobic fitness as well as jumping performance. Furthermore, these results highlight the necessity of personalized training strategies tailored to specific performance goals.
